# The chromosome-level genome assembly of the slug *Deroceras laeve* facilitates its use as a comparative model of regeneration

**DOI:** 10.1093/g3journal/jkaf164

**Published:** 2025-07-30

**Authors:** Jerónimo R Miranda-Rodríguez, Javan Okendo, Wilbert Gutiérrez-Sarmiento, Tobiáš Ber, Josef Pasulka, Kateryna Nemesh, Aranza Torrado-Tapias, Emilio Ortiz-Ávila, Obed Ramírez-Sánchez, Carlos Lozano-Flores, Luis F Hernández-Ramírez, Maribel Hernández-Rosales, Petr Svoboda, Shawn M Burgess, Alfredo Varela-Echavarría

**Affiliations:** Department of Developmental Neurobiology and Neurophysiology, Instituto de Neurobiología, Universidad Nacional Autónoma de México (UNAM), Querétaro, Querétaro 76230, México; Translational and Functional Genomics Branch, National Human Genome Research Institute, Bethesda, MD 20892, United States; Department of Developmental Neurobiology and Neurophysiology, Instituto de Neurobiología, Universidad Nacional Autónoma de México (UNAM), Querétaro, Querétaro 76230, México; Laboratory of Epigenetic Regulations, Institute of Molecular Genetics of the Czech Academy of Sciences, 142 20 Prague 4, Czech Republic; Laboratory of Epigenetic Regulations, Institute of Molecular Genetics of the Czech Academy of Sciences, 142 20 Prague 4, Czech Republic; Laboratory of Epigenetic Regulations, Institute of Molecular Genetics of the Czech Academy of Sciences, 142 20 Prague 4, Czech Republic; Translational and Functional Genomics Branch, National Human Genome Research Institute, Bethesda, MD 20892, United States; Department of Developmental Neurobiology and Neurophysiology, Instituto de Neurobiología, Universidad Nacional Autónoma de México (UNAM), Querétaro, Querétaro 76230, México; CINVESTAV Unidad Irapuato, Irapuato, Guanajuato 36821, México; Department of Developmental Neurobiology and Neurophysiology, Instituto de Neurobiología, Universidad Nacional Autónoma de México (UNAM), Querétaro, Querétaro 76230, México; CINVESTAV Unidad Irapuato, Irapuato, Guanajuato 36821, México; CINVESTAV Unidad Irapuato, Irapuato, Guanajuato 36821, México; Laboratory of Epigenetic Regulations, Institute of Molecular Genetics of the Czech Academy of Sciences, 142 20 Prague 4, Czech Republic; Translational and Functional Genomics Branch, National Human Genome Research Institute, Bethesda, MD 20892, United States; Department of Developmental Neurobiology and Neurophysiology, Instituto de Neurobiología, Universidad Nacional Autónoma de México (UNAM), Querétaro, Querétaro 76230, México

**Keywords:** mollusk, Stylommatophora, Hi-C, PacBio, Genome Assembly, cognate chromosomes

## Abstract

The genome of the *land slug Deroceras laeve* was sequenced, assembled up to the chromosome level, and annotated for non-coding RNAs and protein-coding genes. Due to the small size of this pulmonate species, ease of laboratory culture, cosmopolitan distribution, as well as recently released anatomical and histological resources, this genomic resource creates new opportunities for the investigation of the largely unexplored mechanisms of regeneration in *mollusks*. Moreover, it also makes this slug an attractive model for functional genomics and evolutionary biology.

## Introduction

When comparing gene gain and loss in bilaterian evolution, *spiralians* in general and within them, *mollusks*, offer a crucial point of comparison. They represent a group different from *ecdysozoa*, the group that includes the conventional model organisms the *fruit fly Drosophila melanogaster* and the *nematode Caenorhabditis elegans*, and from *deuterostomes*, which include all *vertebrates* (Simakov et al. 2013; Luo et al. 2018).

The first *spiralian* and *mollusk* genomes that were sequenced tended to be shorter than 600 Mb and showed genomic characteristics closer to *deuterostomes* than to other *spiralians*, such as the *planaria Schmidtea mediterranea* that underwent extensive gene loss (Zhang et al. 2012; Simakov et al. 2013; Guijarro-Clarke et al. 2020; Liao et al. 2023). *Spiralians* conserve many ancient bilaterian genes, particularly those relevant to development (Luo et al. 2018). However, conserved gene networks related to the extensive regenerative capacity of various *spiralian phyla* remain to be determined (Bely et al. 2015). In particular, little research has been done on the cellular and molecular basis of the great capacity of *mollusks* to regenerate damaged or lost tissues, a fact that has been known since the 18th century and supported by more recent research (Tsonis and Fox 2009; Lozano-Flores et al. 2024). Important insights into the evolution of this process have recently been gained from studies on other *invertebrate* and *vertebrate* species (Fossati et al. 2013; Accorsi et al. 2024). Regarding regeneration, the addition of another model that is more evolutionarily diverse will allow researchers to identify both common aspects and characteristics that could be specific to anything from a phylum down to the individual species. Understanding these aspects of regeneration is essential if we want to learn to control such process.

Two of the best studied organisms from the perspective of regeneration are *axolotl* and *planaria*. The genome of *axolotl* is relatively large and full of repeats and long introns (Nowoshilow et al. 2018), while *planaria* has a compact genome with many lost genes (Simakov et al. 2013). Planarian genome dynamics is notable for frequent inversions and interchromosomal rearrangements within a genus (e. g. *Schmidtea*) and even in haplotypes within a species (Ivanković et al. 2024). This complicates the analysis of genomic regulation and the application of these findings to other *metazoans*. Therefore, new high-quality genomes of regeneration-capable organisms such as *mollusks* are needed (Parey et al. 2024), along with knowledge of how their tissues and organs are restored after injury (Davison and Neiman 2021).

Abundant human commensals have traditionally made the best model organisms (Matthews and Vosshall 2020). The *slug D. laeve* is an ubiquitous invasive species that is an easy-to-study and easy-to-rear laboratory animal, as well as an agricultural pest (Gupta et al. 2024). Since *slugs* lack a hard shell, it is possible to study the regeneration of essentially any organ, including the nervous system. Recently, we made widely available a histological atlas that demonstrates the feasibility of employing *D. laeve* as a model organism in regeneration (Lozano-Flores et al. 2024). Here, we present a high-quality annotated chromosome-level assembly of its haploid genome as a significant addition to our efforts to develop it as a study model.

## Materials and methods

### Animals

*Slugs* of the *D. laeve* INB-UNAM strain were obtained as described previously (Lozano-Flores et al. 2024) and used for PacBio and Hi-C genomic sequencing and for the generation of RNA-seq data of the body wall.

For isolation of long and small RNA for microRNA and piRNA identification, *D. laeve* slugs were provided by Heike Reise and John M.C. Hutchinson (Senckenberg Museum für Naturkunde Görlitz) from a reproducing laboratory colony initiated with animals collected at Frankfurt and Glasgow Botanical Gardens. The animals were kept at ${15\;}^{\circ }$C in plastic containers (Plastium GmbH, Nr. 41128-0101) lined with wet tissue and fed iceberg lettuce, oats, and cat food once or twice a week.

### Library preparation and sequencing

High molecular weight DNA was extracted using the Circulomics Nanobind Tissue Big DNA Kit Cat #6505-6505. Briefly, approximately 100 mg of tissue from the body wall of an adult *D. laeve* individual were minced and homogenized using a chilled pestle with 750 μL of cold Buffer CT. The homogenate was then pelleted by centrifugation at 3,000 × *g* for 5 min at ${4\;}^{\circ }$C. The supernatant was discarded and the pellet was resuspended in 1 mL of Buffer CT, followed by a second centrifugation. After centrifugation, the pellet was dislodged by vortexing, resuspended in a mixture of Proteinase K and Buffer CLE3, and thoroughly mixed by pipetting. The solution was incubated in a ThermoMixer at ${55\;}^{\circ }$C and 900 rpm for 30 min. Following incubation, RNase was added, and the sample was incubated again at the same temperature and rotation speed for an additional 40 min. After incubation, 60 μL of Buffer SB was added, and the mixture was vortexed. The sample was centrifuged at 10,000 × *g* for 5 min at room temperature (RT). The supernatant was carefully transferred (300 μL) to a new 1.5 mL microtube. Fifty microliters of Buffer BL3 was added and the mixture was inverted 10 times, ensuring a thorough mixing. A Nanobind disk was placed in the lysate, followed by the addition of 350 μL of isopropanol. The mixture was mixed at 20 rpm for 15 min at RT. The tube was then transferred to a magnetic base and the supernatant was carefully discarded. Next, 500 μL of Buffer CW1 was added, and the tube was removed from the magnetic base and inverted 4 times. The tube was returned to the magnetic base, and the supernatant was discarded. This wash step was repeated with an additional 500 μL of Buffer CW1. Following this, 500 μL of Buffer CW2 was added, and the tube was removed again from the magnetic base and inverted 4 times. The tube was placed back on the magnetic base, and the supernatant was discarded. The CW2 wash step was repeated once more. Next, 75 μL of Buffer EB was added directly to the Nanobind disk, and the solution was incubated for 10 min at RT. After incubation, DNA was collected and transferred to a new 1.5 mL microtube. The sample was pipetted several times to ensure complete homogenization and to break up any remaining “jellies.” The DNA was allowed to solubilize overnight before proceeding with downstream analysis.

The Pacific Biosciences protocol “Preparing HiFi SMRTbell Libraries using the SMRTbell Express Template Prep Kit 3.0” was used to create 2 libraries from 15 micrograms of genomic DNA. The Megarupter (Diagenode) was used for shearing. The libraries were pooled for size selection using a Sage ELF (Sage Science). Fractions containing fragments >17 kb were pooled for sequencing. Library BCR0004_1 (topUp) was run on two 8M SMRTCells using version 2.0 sequencing reagents. Sequencing was performed on a Sequel IIe sequencer (Pacific Biosciences) running instrument control software version 11.0.0.144466 and a movie collection time of 30 h per SMRTCell with adaptive loading. CCS/HiFi reads were generated on-instrument (running primary software version 11.0.0.144466) and then imported into PacBio SMRTLink (running version 11.0.0.146107). Fastq files were generated from the hifi_reads.bam files using the pb_export_ccs workflow.

For Oxford Nanopore sequencing, genomic DNA libraries were created from ultra-high molecular weight DNA from tissue using the SQK-ULK114 sequencing kit and run on a PromethION with MinKNOW software version MinKNOW 24.02.19. Each library was loaded onto a FLO-PRO114M flow cell and run for 72 h or more with 2 subsequent loadings at 24-h intervals. Base calling was performed using Dorado 7.3.11.

For Hi-C sequencing, 6 adult individuals weighing between 300 and 500 mg were dissected to expose the visceral mass, the digestive tract was slit open throughout its length, its contents were emptied, and the entire mass was washed 3 times in PBS. The body wall and the washed visceral mass were minced into small pieces and fixed in 1% PFA for 20 min at approximately ${25\;}^{\circ }$C. Glycine granules were added to a final concentration of 125 mM and the samples were further incubated at RT for 15 min. The samples were snap-frozen in liquid nitrogen, ground with a mortar to a fine powder, and stored frozen. The frozen tissue was shipped to Phase Genomics (Seattle, WA) for genomic DNA extraction and Hi-C sequencing using the Proximo Kit protocol.

### Genome assembly

We performed the genome assembly with Verkko (version 1.4.1) (Rautiainen et al. 2023), using PacBio HiFi reads, producing a 1.8 Gb final assembly. To resolve the tangles in the assembly, we aligned ONT reads to the Verkko-generated assembly graph using GraphAligner (Rautiainen and Marschall 2020). Since Verkko output is homopolymer compressed, we first converted ONT fastq file to fasta then applied homopolymer compression to the ONT reads using “seqtk hpc” command. The resulting alignment is recorded in the “graphaligner.gaf” file, we manually inspected to identify the correct ONT reads traversals across the tangle regions. These validated paths were then extracted and provided to Verkko and an input for gap patching. The following command was used to conduct the alignment:

GraphAligner -t 24 -g assembly.homopolymer-compressed.gfa

-f ONThomopolymerCompressed.fasta -a graphaliner.gaf

--seeds-mxm-windowsize 5000 --seeds-mxm-length 30

--seeds-mem-count 10000 --bandwidth 15

--multimap-score-fraction 0.99 --precise-clipping 0.85

--min-alignment-score 5000 --hpc-collapse-reads

--discard-cigar --clip-ambiguous-ends 100

--overlap-incompatible-cutoff 0.15 --max-trace-count 5

--mem-index-no-wavelet-tree

An example of a read spanning a tangle in the assembly appears as follows:

1880c0f9-b3d2-4e41-a606-b3cf4de00428 133770 59 130460 +

<utig4-577<gapont1-1-len-46542-cov-3>utig4-1953\

98221777 98091240 98221777 0 0 60

In this example, the read (*1880c0f9-b3d2-4e41-a606-b3cf4de00428*) aligns across 2 utigs, utig4-577 and utig4-1953, indicating a correct traversal that is used to patch the gap. The read spanning the gap is 46,542 base pairs (46 kb) long. We then prepared a tab delimited “paths.gaf” file as follows: *Chr1 ¡utig4-577¡gapont1-1-len-46542-cov-3¿utig4-1953 Hap*. Run Verkko again using –paths “path.gaf” together with the original verkko assembly results directory. The resulting assembly will have all the gaps and tangles patched.

We also observed that the single nucleotide polymorphism (SNP) rate was extremely low based on a K-mer analysis (not shown), probably due to the fact that the laboratory colony was derived from a few individuals, hence creating a genetic bottleneck. Accordingly, since there was not enough SNP data to phase haplotypes, we treated this genome as haploid and deriving from a nearly inbred line.

To generate chromosome-length scaffolds, the Hi-C reads were aligned to the draft genome using the juicer v1.6 pipeline (Durand et al. 2016b). The open access scripts from juicer were modifed to accommodate the restriction enzymes used for Hi-C library preparation DpnII (GATC), DdeI (CTNAG), MseI (TTAA), and HinfI (GANTC). The 3D-DNA pipeline v180922 was used to anchor, order, and orient the contigs of the draft assembly (Dudchenko et al. 2017). Only contigs with a length >13 kb were used, and the assembly was further reviewed and polished using Juicebox Assembly Tools v2.17 (Durand et al. 2016a; Dudchenko et al. 2018).

### Mitogenome identification

A reference mitochondrial genome was aligned with LastZ against each scaffold in the *D. laeve* genome assembly. The only match was scaffold 1,563. The annotated mitogenome was visualized and aligned to scaffold 1,563 in SnapGene software (v8.0.1). An annotated mitochondrial genome containing the specific variants of our samples was generated using the function Replace Original with Aligned within SnapGene. For each mitochondrial coding gene, both the reference and the variant CDS were extracted and aligned with MAFFT v7.299b (Katoh et al. 2002). The alignments were analyzed for dN/dS ratios using codeml from paml v4.9d (Yang 2007) with the parameter icode = 4 to introduce invertebrate mitochondrial codons.

### RNA isolation

The slugs were killed in ice cold ethanol. Juvenile slugs or dissected tissues were washed with PBS, homogenized in Qiazol lysis reagent (Qiagen), and total RNA was isolated by Qiazol-chloroform extraction and ethanol precipitation (Toni et al. 2018). Furthermore, body wall tissue was also used for RNA extraction with the RNeasy kit (Qiagen).

### Long RNA sequencing

After isolation, RNA quality was verified by electrophoresis on 1% agarose gel, and RNA concentration was determined with the Qubit Broad Range Assay (Invitrogen). Poly-A RNA was purified using the ${\mathrm{Dynabeads}}^{\mathrm{TM}}$ mRNA Purification Kit (Invitrogen) according to the manufacturer’s protocol. Poly-A RNA concentration was determined with the Qubit Broad Range Assay (Invitrogen). Long RNA libraries were prepared with the NEBNext Ultra II Directional RNA Library Prep Kit for Illumina (New England Biolabs) according to the manufacturer’s protocol with 6 to 8 cycles of PCR amplification. Libraries were analyzed in the Agilent 2100 Bioanalyzer and sequenced in 138-nucleotide single-end reads using the Illumina NextSeq2000 platform. Furthermore, paired-end RNA libraries from body wall tissue were generated using TruSeq Stranded mRNA Library Prep and sequenced using 2x75 NextSeq 550 High output platform.

### Small RNA sequencing

After isolation, RNA quality was verified by electrophoresis on 1% agarose gel, and RNA concentration was determined using the Qubit Broad Range Assay (Invitrogen). Small RNA libraries were prepared with the NEXTFLEX Small RNA-Seq Kit v4 for Illumina (Revitty) according to the manufacturer’s protocol with 16 to 18 cycles of PCR amplification. For clean-up and size selection, the libraries were separated on a 2.5% agarose gel using 1× lithium borate buffer and visualized with ethidium bromide. The 150–160 base pair fraction was cut from the gel, and DNA was isolated using the MinElute Gel Extraction Kit (Qiagen). The final libraries were analyzed on an Agilent 2100 Bioanalyzer and sequenced in 138-nucleotide single-end reads on the Illumina NextSeq2000 platform.

### Mapping of small RNA-seq data onto the genome

The small RNA-seq reads were trimmed with CUTADAPT v2.5 (Martin 2011) and mapped to the *D. laeve* genome using STAR (Dobin et al. 2013) v2.7.10b. Mapped reads were counted using a homemade R script. Only reads with lengths 18-32 nt were selected from the small RNA-seq data.

### miRNA annotation

Mapped reads aligned to the reference genome were subjected to miRNA prediction using MIREAP with default parameters (https://github.com/liqb/mireap). The algorithm predicted 846 putative 5p and 3p miRNAs. Upon removing miRNAs containing repeat sequences or having very low expression, genomic loci of 674 were individually inspected and classified into 4 categories: *high confidence* (matching sequence of a miRNA already annotated in miRGeneDB 3.0 (Clarke et al. 2025) or the main strand was abundant, its 5’ end was well-defined, the passenger was typically visible, and a pre-miRNA-like stem loop could be predicted), *moderate confidence* (the main strand was expressed <100 RPM in all samples, <20 RPM for miRNAs with 2 to 10 nucleotide sequence identity, it had its 5’ end clearly defined and a pre-miRNA-like stem loop could be predicted), *ambiguous* (very low expression or poorly defined 5’ end of the main strand or stem loop could not be predicted), *artifact* (repeat-derived, low complexity or overlapping with a highly abundant piRNA). Subsequently, the list of high and moderate confidence miRNAs was refined to annotate both 5p and 3p strands of 178 precursor loci by comparing with miRNAs annotated in miRGeneDB 3.0 (Clarke et al. 2025) for the bivalve Pacific oyster (*Magallana gigas*, 135 genes, 59 families) and the gastropods owl limpet (*Lottia gigantea*, 80 miRNA genes, 53 families) and red abalone (*Haliotis rufescens*, 83 genes, 55 families) (Supplementary Tables S1 and S2).

### piRNA annotation

The mapped reads (27–30nt) were subjected to *de novo* annotation of piRNA clusters with the default parameters of the piRNA cluster builder (piCB), a toolkit for piRNA analysis available online (Konstantinidou et al. 2024) (https://github.com/HaaseLab/PICB). The final list of piRNA clusters was manually curated, and adjacent clusters were merged if they were oriented in the same direction and were <5 kb apart. This produced 170 clusters, which were ranked and annotated according to the number of piRNAs they produce (Supplementary Table S3).

### Repeat identification and annotation

To annotate repeats, we downloaded a repeat library from Dfam 3.8 (Storer et al. 2021) by selecting the taxon Spiralia that includes descendants and ancestors but does not include uncurated repeats. We identified *de novo* repeat elements using RepeatModeller v2.0.3 (Flynn et al. 2020) with LTR structural analysis enabled. RepeatModeller identified 1705 families, of which 1109 could not be classified by the RepeatClassifier module. These unidentified families were assigned a repeat category based on the highest TEclass score (Abrusán et al. 2009). As a complement, we used HiTE v3.2, a novel fully automated pipeline for TE detection and annotation that prioritizes the identification of full-length “intact” transposon copies (Hu et al. 2024).

Interspersed repeats were used to hardmask the genome, and a further round of tandem repeat finder was run to identify low-complexity sequences (Benson 1999).

To identify tRNA and rRNA loci, we used tRNAscan-SE v2.0.12 (Seemann 2013) and barrnap v0.9 (Chan et al. 2021).

### Genome annotation

We generated gene models with BRAKER (Stanke et al. 2006, 2008) using metazoan proteins and transcriptome hints. In parallel, we built a *de novo* transcriptome with paired end RNAseq reads from body wall tissue and a genome-guided transcriptome assembly from 3 single end RNAseq libraries from whole juveniles, ovotestes, and head. We combined the 2 transcriptomes into PASA (Program to Assemble Spliced Alignments) assemblies by spliced alignment of the transcripts with the genome (PASA v2.5.3) (Haas et al. 2003). Using EVidenceModeller v2.1.0 (Haas et al. 2008), the BRAKER gene models were combined with the PASA assemblies giving more weight to those assemblies with a predicted ORF longer than 125 aa. The resulting gene models were compared with the PASA assemblies to add UTRs, join split genes, and correct coding sequences according to evidence from the transcriptome. To assess proteome completeness, the longest ORF from each gene model was selected. This non-redundant proteome was compared with the metazoa_odb10 and mollusca_odb10 datasets of BUSCO v5.4.2 (Manni et al. 2021). The proteome was also compared with the Lophotrochozoa clade in the OMA database using the OMArk web service v0.3.0 (Nevers et al. 2022). This proteome was compared with KAAS (Moriya et al. 2007) and the eggNOG mapper (Cantalapiedra et al. 2021), and the results for each protein were used to annotate the corresponding gene model. To identify putative paralogs, we used the ortholog output from eggNOG. We compared the set of orthologs for each gene with the set of all other genes and established a link between genes with jaccard similarity values (intersection divided by the union) higher than a threshold.

### Whole genome alignment and intrasynteny

For alignment, the interspersed and tandem repeats *Deroceras laeve* genome were hardmasked. Lastz 1.04.15 was used to align each *D. laeve* scaffold separately against the whole genome of *D. laeve*, in the case of self-alignment, or the genome of *Achatina fulica*. For self-alignment, a previous self-masking round with lastz was performed to softmask the sequences appearing more than 3 times in an alignment, thus excluding them from the initial seeds but not from extension.

In this work, we define a cognate for each chromosome *i* in the same species as follows:


$$Cognate(i)=\max_{\;j\neq i}\frac{\ell_{i,j}}{L_{i}-M_{i}}$$

Where $\ell_{i,j}$ is the number of bases of chromosome *i* that are covered by alignment to chromosome *j*, $L_{i}$ is the total length of chromosome *i* and $M_{i}$ the number of bases masked by tandem and interspersed repeats in chromosome *i*. For each chromosome *i*, the chromosome that yields the maximum number is its “cognate”.

Homologies between the 31 chromosomes from *D. laeve* and *D. lasithionense* were obtained by running ragtag 2.1.0 with the option scaffold using the *D. lasithionense* genome as reference. The original 31 chromosome-sized HiC scaffolds in our assembly take the Chromosome numbering and strand as the *D. lasithionense* genome.

### Genomic comparative

Gene orthogroups (GOs) were inferred with the Reconstruction of Evolutionary Histories tool (REvolutionH-tl, v1.2.1) (Ramírez-Rafael et al. 2024) from the non-redundant *Deroceras laeve* proteome described above and these 20 molluscan species: Argopecten irradians, Elysia chlorotica, Octopus vulgaris, Arion vulgaris, Elysia crispata, Patella vulgata, Biomphalaria glabrata, Elysia marginata, Pinctada imbricata, Bulinus truncatus, Lymnaea stagnalis, Plakobranchus ocellatus, Crassostrea virginica, Mytilus galloprovincialis, Pomacea canaliculata, Octopus bimaculoides, Ruditapes philippinarum, Dreissena polymorpha, Octopus sinensis, and *Sepia pharaonis*. A UPGMA phylogenetic tree was constructed on Molecular Evolutionary Genetics Analysis (MEGA,11.0.13) setting *Octopus sp.* as the outgroup. The tree was calibrated using information from the timetree (Kumar et al. 2022). We then used the reconstructed UPGMA tree to generate the reconciliation tree along with its summary information, corresponding to Step 5 of REvolutionH-tl. The significantly expanded and contracted gene families were also identified by computational analysis of the gene family evolution tool (CAFE, v4.2.1) based on the 22 mollusk species. The heatmap of the main gene ontology for expanded and contracted families was constructed in Rstudio v4.4.1 with *patchwork* v1.3.0. Next, synteny block identification between the *D. laeve* and *D. lasithioniense* pair was performed using ntSynt (v1.0.2) with default parameters. Then, *D. laeve* chromosomes were reordered and renamed according to the detected genome synteny with *D. lasithioniense* chromosomes. Similarly, the identification of synteny blocks was carried out for the *D. laeve*/*A. fulica* and *D. laeve*/*A. vulgaris* pairs. Subsequently, we normalized the input chromosome strands based on the target assembly (*D. laeve*) using ntSynt-viz (v1.0.0). Finally, we used NGenomeSyn (v1.41) to generate visualizations, where the *A. fulica* and *A. vulgaris* chromosomes were reordered based on the detected genome synteny with *D. laeve*.

### Data analysis methods

A step by step guide that includes scripts and options selected for each of the bioinformatics procedures is available at: https://github.com/jerolon/Dlaeve_genome/.

## Results and discussion

### Assembly statistics

**Fig. 1.**
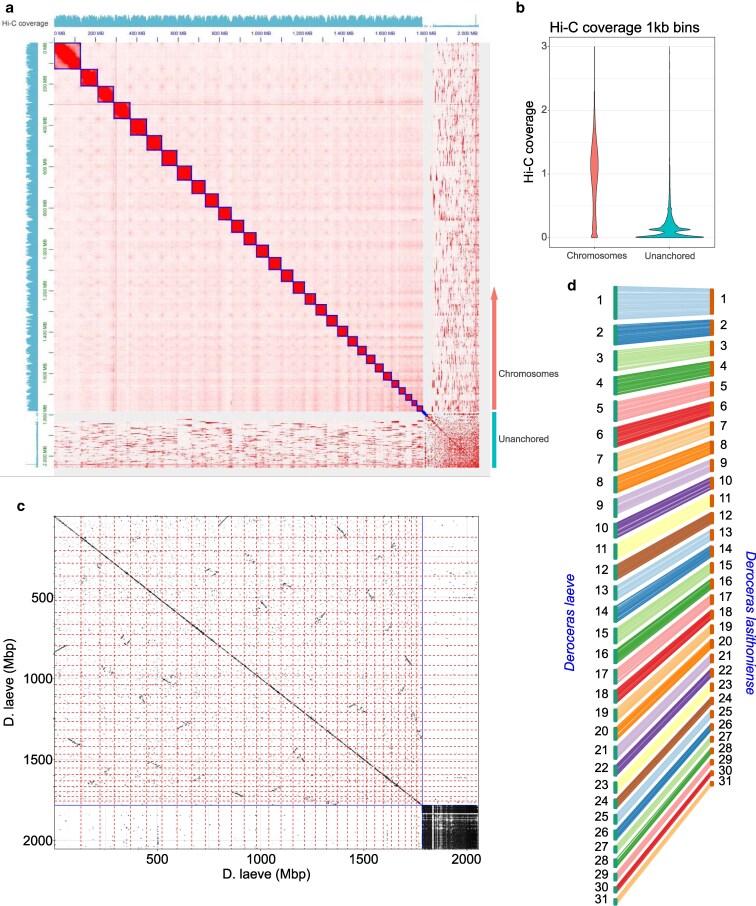
Hi-C-guided chromosome level scaffolding. a) Heatmap of the normalized proximity signal from the Hi-C analysis. Blue line squares enclose the scaffolds that are defined by an enriched proximity signal along the diagonal. Scaffolds are arranged in descending order by length. The Hi-C coverage is shown along the *y* and *x* axes. b) Hi-C coverage markedly differs between the main assembly and the unplaced scaffolds. c) Whole genome self-alignment of the *D. laeve* assembly showing long stretches of inter-chromosomal synteny as off-diagonal alignments. The vertical and horizontal blue lines indicate the boundary between *D. laeve* chromosomes and unplaced scaffolds. d) Conserved one-to-one synteny between the 31 chromosomes of 2 *Deroceras* gastropods.

We assembled 2,051,930,631 base pairs distributed between 2,212 contigs using PacBio HiFi reads, which were further assembled into 31 chromosome-sized scaffolds with Hi-C data (Fig. 1a). The contig N50 was 2,557,437 and the L50 was 243, and after Hi-C assembly, the scaffold N50 was 59,470,895 with an L50 value of 14. This chromosome number and genome size are within the range reported for the Order *Stylommatophora* and Family *Limacidae* (Thiriot-Quiévreux 2003). The chromosomes comprise approximately 1.78 Gb of sequence. The rest, approximately 200 Mb, were assembled into smaller scaffolds that could not be reliably placed on chromosomes due to low Hi-C coverage (Fig. 1a, b).

BUSCO scores show a high completeness of the assembly. A comparison to metazoa_odb10 returned a completeness score of 93.5% [S: 83.3%, D: 10.2%], F: 1.2%, M: 5.3%, n: 954. A phylogenetically closer comparison to mollusca_odb10 had 88.1% completeness [S: 71.8%, D: 16.3%], F: 0.4%, M: 11.5%, n: 529.

A self-alignment of the *D. laeve* assembly also reveals broad intrachromosomal synteny (Fig. 1c), possibly the result of the previously described whole genome duplication (WGD) in the ancestors of *Stylommatophora* (Chen et al. 2022). Unplaced scaffolds from *D. laeve* show little similarity to the chromosomes but high similarity to other unplaced scaffolds, suggesting that they are part of tandem repeat fragments, which could be too long or missing identifying SNVs for the Hi-C data to resolve effectively (Fig. 1c). Furthermore, alignment of the genome (including unplaced scaffolds) with the assembled genome of *Achatina fulica*, a *stylomatophoran gastropod* containing the same number of chromosomes (Guo et al. 2019) reveals conserved synteny tracts for most chromosomes. Only a few unplaced scaffolds from *D. laeve* align with the *A. fulica* assembly (Supplementary Fig. S1a, below the blue line), and the pattern again suggests repetitive elements.

We also compared our *D. laeve* assembly with the genome of another member of the same genus, *D. lasithionense*. We found a one-to-one correspondence between these genomes when matching synteny tracts. Both species possess 31 chromosomes, although their size varies between species. To facilitate future comparative studies, however, we named the *D. laeve* chromosomal scaffolds to match their *D. lasithionense* homologs (Fig. 1d, Supplementary File S1). This comparison with a closely related genome that was independently assembled is further evidence of the quality of both assemblies.

Syntenic tracts are also conserved between *D. laeve* and the more distantly related stylomatophorans *A. fulica* and *Arion vulgaris*. The largest chromosome 1 is particularly well conserved between these 3 species. Nevertheless, although they all have 31 chromosomes, they show a history of rearrangements, splits, and fusions. Notable is chromosome 3 of *D. laeve* that is split both in *A. vulgaris* (chr21 and 22) and *A. fulica* (chr20 and 26) (Supplementary Fig. S1b). In contrast, chromosome 2 of *A. vulgaris* appears to correspond to chr5 and 12 of *D. laeve* and chr14 and 19 of *A. fulica*.

The unanchored scaffolds (200 Mb) were also classified according to their possible phylogeny using Kraken (Wood and Salzberg 2014), by the percentage of bases covered by RNA-seq reads, and by the percentage of repeats which are described in detail in later sections. Those classified as Eumetazoa constitute 55 Mb in length. The vast majority of them, and those classified as *prokaryotic, fungi, or plant*, possess high repeat content and low RNA-seq coverage (Supplementary Fig. S1c, d).

Furthermore, we searched for tRNA and rRNA loci within the unplaced scaffolds. The Eukaryotic scaffolds have a higher content of rRNA. Meanwhile, the biggest unplaced scaffolds (HiC_Scaffold_32 - 47), that are all predicted to be bacterial by kraken, contain 5 to 20 rRNA loci (Supplementary Fig. S1e). At the same time, these bacterial scaffolds have tRNA loci that carry a greater variety of aminoacids, while the Eukaryotic scaffolds carry very few of them (Supplementary Fig. S1f, File S2).

These findings convinced us that most sequences that can be reliably placed in chromosomes are correctly included in the assembly. Unplaced scaffolds probably correspond to repeats, including rRNAs, telomeric, and centromeric sequences common to many chromosomes. Some scaffolds, specially those classified as bacterial by kraken, appear to represent contamination from the internal or external slug microbiome or uncharacterized symbionts. Hence, these scaffolds were removed from the assembly.

### Mitochondrial genome

**Fig. 2.**
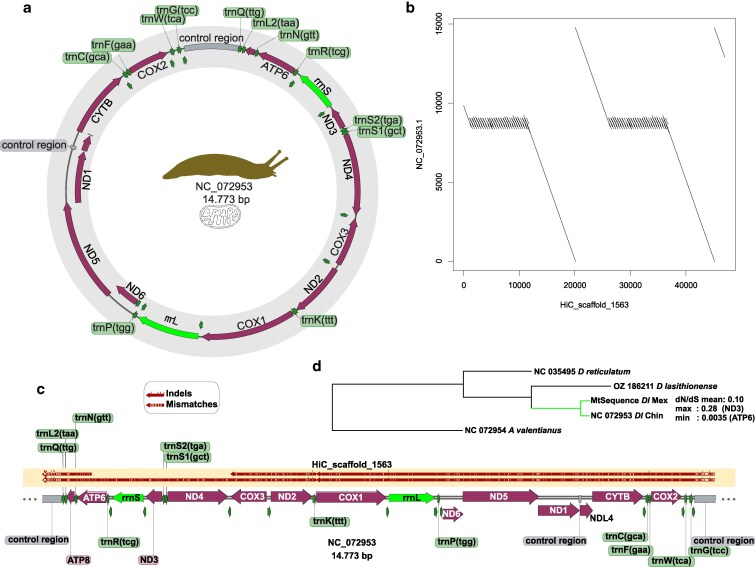
Map of *Deroceras laeve* mitochondrial genome. a) Circular map of the mitochondrial genome with features annotated. The sense of arrows indicate the direction of transcription. b) Alignment of NC_072953 to Hi-C scaffold 1563. c) Linear map of the mitochondrial chromosome. Gaps in the segments in the shaded box represent mutations and small notches, or indels. d) Phylogenetic reconstruction with full mitochondrial sequences from the limacids *D. lasithionense, D. reticulatum*, *Ambigolimax valentianus*, a recently reported *D. laeve* sequence (Dl Chin) and the one obtained in this study (Dl Mex).

Further examination of scaffold 1,563, an outlier with low repeat content and high coverage of RNA sequence, classified as eumetazoan by Kraken (Supplementary Table S4, Fig. S1c), revealed that it corresponds to the mitochondrial genome. We used as a reference the sequence with accession number NC_072953 from the NCBI Organelle Refseq Project. The circularized mitochondrial genome of *D. laeve* is 14,773 bp and is annotated with protein-coding genes, tRNAs, rRNAs, and 2 control regions (Fig. 2a). An alignment of NC_072953 to the whole genome assembly matches exclusively to scaffold 1,563. The alignment pattern is evidence of the concatemerization of 2 almost complete full-length units (Fig. 2b, c). The mitochondrial sequence obtained by Hi-C is almost perfectly continuous, except for one of the control regions that is tandemly repeated in the scaffold. The concatemerized copies within scaffold 1,563 are identical; therefore, we reconstructed a mitochondrial sequence for our colony by replacing all differences in the reference with our sequence. Comparison of all mitochondrial Open Reading Frames (ORFs) present a signature of purifying selection (mean dN/dS ratio of 0.10). Cytochrome c oxidase I (COX1) and NADH dehydrogenase (ND6) were excluded from the analysis because they did not have non-synonymous mutations. Phylogenetic reconstruction with the complete mitochondrial genomes of this and other limacid species, including *Deroceras reticulatum* and *Deroceras lasithionense* (Table 1), revealed that the sequence of our colony aligned closest to a *D. laeve* isolate from China, confirming its identity and revealing its probable origin (Fig. 2d, Supplementary File S3).

**Table 1.** Accession codes of reference sequences used in this study.

Species	Accession number	Level	Number
*Argopecten irradians*	GCA_041381155.1	Chromosomes	16
*Biomphalaria glabrata*	GCF_947242115.1	Chromosomes	18
*Bulinus truncatus*	GCA_021962125.1	Scaffolds	523
*Crassostrea virginica*	GCF_002022765.2	Chromosomes	10
*Dreissena polymorpha*	GCF_020536995.1	Chromosomes	16
*Elysia chlorotica*	GCA_003991915.1	Scaffolds	9,989
*Elysia crispata*	GCA_033675545.1	Contigs	8,107
*Elysia marginata*	GCA_019649035.1	Scaffolds	14,149
*Lymnaea stagnalis*	GCA_964033795.1	Scaffolds	6,640
*Mytilus galloprovincialis*	GCA_900618805.1	Scaffolds	10,577
*Octopus bimaculoides*	GCF_001194135.2	Chromosomes	30
*Octopus sinensis*	GCF_006345805.1	Chromosomes	30
*Octopus vulgaris*	GCA_951406725.2	Chromosomes	30
*Patella vulgata*	GCF_932274485.2	Chromosomes	9
*Pinctada imbricata*	GCA_033119305.1	Scaffolds	767
*Plakobranchus ocellatus*	GCA_019648995.1	Scaffolds	8,647
*Pomacea canaliculata*	GCF_003073045.1	Chromosomes	14
*Ruditapes philippinarum*	GCF_026571515.1	Contigs	15,873
*Sepia pharaonis*	GCA_903632075.3	Contigs	5,642
*Deroceras lasithionense*	GCA_964271515.2	Chromosomes	31
*Arion vulgaris*	GCA_020796225.	Chromosomes	26
*Achatina fulica*	10.5524/100647	Chromosomes	31
*Deroceras laeve*	NC_072953	Mitochondrial Genome	1
*Deroceras lasithionense*	OZ186211.1	Mitochondrial Genome	1
*Deroceras reticulatum*	NC035495	Mitochondrial Genome	1
*Ambigolimax valentianus*	NC072954	Mitochondrial Genome	1

### Repeat content

We employed 2 strategies to annotate and mask repeats. In the first strategy, inspired by a published pipeline (Card et al. 2019), we combined a Spiralia repeat library with our *de novo* library for *D. laeve* produced by RepeatModeller. For the second strategy, we used a fully automated *de novo* annotation pipeline, HiTE that has been reported to excel in identifying full-length transposon copies (Hu et al. 2024). HiTE focuses on specificity and prioritizes “intact” and high confidence repeat elements. We then compared both annotation results and classified the different types of repeats detected (Table 2, Supplementary Tables S5 and S6). We believe the combined strategies provide a lower and upper bounds of repeat content composition (Supplementary File S4).

**Table 2.** Side-by-side comparison of 2 repeat annotation pipelines showing the number of elements and percentage of genomic sequence covered by different classes of repeats.

	DFAM + RepeatModeller		HiTE	
	# of elements	% of sequence	# of elements	% of sequence
SINEs:	295,508	1.83	236,901	1.61
LINEs:	2,062,222	24.11	728,780	13.84
LTR elements:	337,895	4.73	301,846	3.16
DNA elements:	734,111	7.33	981,000	11.12
Unclassified:	71	0	452,329	4.38
Total insterspersed repeats:	–	37.99	–	34.1
Small RNA:	51,436	0.74	23,6901	1.61
Satellites:	2	0.00	2	0.0
Simple repeats:	525,211	3.86	374,619	3.32
Low complexity:	86,093	0.9	56,316	0.8

**Fig. 3.**
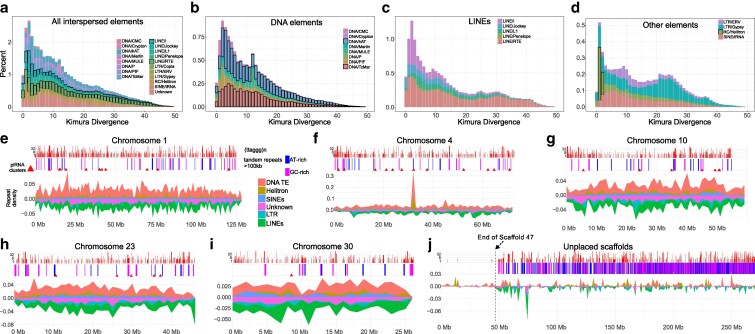
The repeat content landscape occupies 70% of the slug genome. a) General repeat divergence landscape showing the evolutionary distribution of the most common kind of repeats and sub-classifications. b) Repeat divergence landscape of DNA elements. c) Divergence landscape of LINEs. d) Divergence landscape of LTRs, RC/Helitron and SINEs. e–i) Distribution of selected types of repeats in Chromosomes 1, 4, 10, 23, and 30 and j) in Unplaced scaffolds sorted from longest to shortest. For each chromosome, panels show Top: TTAGG repeats found in a given tandem element. Middle: Span of tandem repeats colored by GC enrichment (lighter) or depletion (darker) compared to the genome average GC% (42%). Red triangles show the location of piRNA clusters. Bottom: stacked densities of different classes of interspersed repeats in 1 Mb windows: DNA elements, Rolling Circle/Helitron, LINEs, LTR, SINEs and Unknown classification.

Both strategies agree on an interspersed repeat coverage of 34.1% to 37.99%, of which 1.61% to 1.83% corresponds to short interspersed nuclear elements (SINEs), 7.33% to 11.12% to DNA elements, and 3.16% to 4.73% are long terminal repeat (LTR) elements. The largest discrepancy was in LINE content, likely reflecting that we used a lax threshold for classifying such elements (13.84% to 24.11% of the genome). In either case, LINEs are the largest class of interspersed repeats by genome coverage (Table 2 and Fig. 3a). Therefore, the strategies agree broadly on content and type of repeats: a genome covered overall by approximately 40% interspersed elements, dominated by LINEs of the RTE family, and different DNA elements in second place. A high LINE/SINE content is not common in invertebrates, but it is in line with a positive correlation with the genome size of *Heterobranchia* (Chen et al. 2022). The differences between the 2 pipelines do not appear to be due to identification of sequences as repeat elements but rather to their classification in different subclasses. Thus, the discrepancies highlight the scarcity of well-curated repeat libraries for *lophotrochozoa* and the opportunity for future studies to characterize these transposable families at greater depth.

The repeat divergence landscape shows that most repeats are relatively recent (Fig. 3a–d). DNA elements are dominated by DNA/hAT and DNA/TcMar (Fig. 3b), the latter known for horizontal transfer events (Dupeyron et al. 2020) while the most common LINEs are dominated by low divergence elements such as RTE and LINE-I Jockey, which appear to have undergone a recent expansion (Fig. 3c). We also found a significantly conserved portion of the circular RC/Helitron (Fig. 3d), and lastly, the retrovirus Gypsy shows a recent expansion and a substantial proportion of diverged elements, making it the most abundant LTR (Fig. 3d).

Simple repeats and low-complexity sequences occupy a very small percentage of the repeats reported by RepeatMasker, but a simple examination of the masked sequence revealed the need to further mask tandem repeats. Hence, tandem repeat finder (Benson 1999) was used further augmenting the total repeat bases by 28.39 % of the genome. In total, about 72% of the *D. laeve* genome is covered by interspersed and tandem repeats (Supplementary Fig. S1c, File S4).

The distribution of selected types of interspersed repeated elements and tandem repeats are shown for some examples of chromosomes and for the Unplaced scaffolds (Fig. 3e–i, j), while the distribution in all chromosomes is in Supplementary Fig. S2.

Interspersed repeats are evenly distributed on the chromosomes (Fig. 3e–g, Supplementary Fig. S2), although some highly enriched clusters are evident for DNA elements (Fig. 3e,f) and LTRs (Fig. 3g). In addition, there are long (>100 kb) tandem repeats that are GC-rich, especially at the ends of chromosomes, many containing the characteristic TTAGGG motif of telomeres. Interstitial and subtelomeric TTAGGG repeats, however, are present in all chromosomes (Fig. 3e–i, Supplementary Fig. S2). Such repeats are common in genomes of very different species and could reflect a complex history of chromosome rearrangements (Bolzán 2017).

When it comes to unplaced scaffolds, these sequence segments are characterized by either a very high or very low repeat density. In fact, the longest unplaced scaffolds HiC scaffolds 32 to 47, together comprising 50 Mb (classified as bacterial by kraken) are almost completely devoid of any repeat (Fig. 3j).

### Genome annotation

#### Inference of gene models

**Fig. 4.**
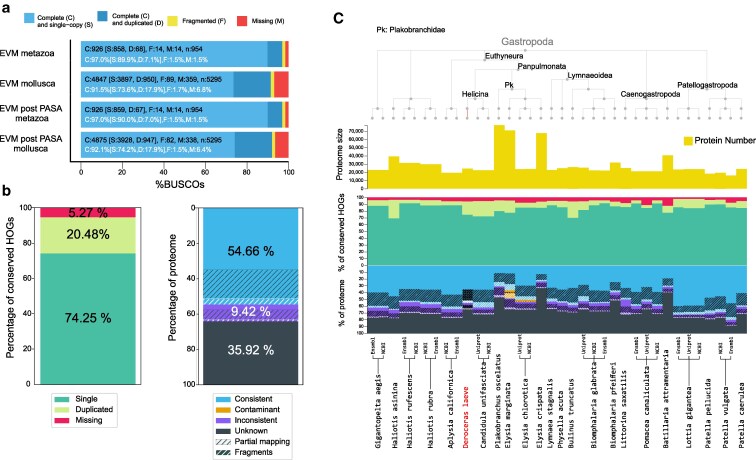
Metrics of *D. laeve* genome annotation quality. a) Benchmarking Universal Single-Copy Orthologs (BUSCO) for the most general (eumetazoan) and the most specific (mollusca) datasets available for the organism. EVM, genes obtained with EvidenceModeller; EVM post PASA, EVM genes enriched with PASA assemblies. b) OMArk results for completeness (percent out of 2,373 lophotrochozoa conserved Hierarchical Orthologous Groups (HOGs) found in *D. laeve* proteome; left) and consistency (percentage of 24,337 *D. laeve* proteins that correspond to a gene family known to exist in the selected lineage; right) c). Comparison of OMArk percentages of *D. laeve* to other gastropod proteomes present in the OMArk database.

We obtained 24,253 high confidence protein coding gene models by combining *de novo* gene predictions with RNA-seq data with EvidenceModeller. The predicted proteome scores 97% and 91% against the metazoan and mollusca BUSCO databases, respectively (Fig. 4a). Gene annotation scores 94.77% for completeness against the lophotrochozoan gene complement in the independent benchmark OMArk database (Fig. 4b). This score and the number of proteins are comparable to other gastropod proteomes present in OMArk. Interestingly, *D. laeve* possesses a higher number of duplicated proteins, which is a feature shared with *Candidula unifasciata* (Fig. 4b, c), the only other stylomatophoran in the database and no evidence of contamination was detected in the predicted proteome. Genes classified as Unknown (35.92%) are those that do not have similarity to known gene families and thus represent orphans. The evidence against them being erroneous gene models is that the proportion of the proteome taxonomically unclassified seems typical for *gasteropod* and *lophotrochozoan* annotations in the OMArk database (Fig. 4c and data not shown). Therefore, the large number of unidentified genes is probably due to the sparsity of functional genetic studies in these species.

EvidenceModeller returns only one CDS per gene without untranslated regions. We next enriched the EVM/Braker annotated genes with PASA assemblies to add 5’ UTR and 3’ UTR regions to gene models supported by RNA-seq experimental evidence, putative low-confidence protein-coding genes supported only by RNA-seq data, and splicing isoforms for the existing gene models. The number of gene models in this PASA-enriched annotation increased slightly to 24,337 and the number of total transcripts to 33,019. The number of mRNAs with both 5’ and 3’ annotated UTRs is 15,045. This enriched database is marginally better in terms of BUSCO completeness than the EVM models alone: 97% and 92.1% in the BUSCO databases of metazoan and mollusks, respectively. The addition of 3’ UTR regions and splicing variants supported by multiple RNA-seq libraries is an important resource for the design of *in situ* hybridization probes or single-cell transcriptomics in this organism and, therefore, future work will be done with the PASA-enriched annotation (Supplementary File S5).

The identified transcripts have an average exon number of 10.1 and an average intron number of 9.1. The exons are 258 base pairs long on average, and the mean intron length is 4,250 base pairs. The percentage of the genome covered by genes is 41.2%, while the fraction covered by coding sequences in exons is 2.9%. Therefore, the non-coding component of this genome is comparable to *vertebrates*. A proposed benchmark for the evaluation of a *de novo* annotation is the ratio of monointronic to multiexon transcripts (Vuruputoor et al. 2023). The number of single exon transcripts in the annotation is 4,410. Therefore, the ratio is (4,410 monoexonic transcripts)/(33,019 total transcripts - 4,410) = 15.41%, which is within the range for eukaryotes and even for vertebrates (Jorquera et al. 2016).

### Protein annotation and gene family evolution

To infer gene function and assign possible orthologs, we compared the proteome with the eggNOG-Mapper and KAAS Mapper databases. In addition, we subsequently performed a blast search against RefSeq proteomes from mollusks. Our annotation includes putative gene names, functions, Gene Ontologies (GOs), and PFAM domains (Supplementary File S5).

When looking at Kyoto Encyclopedia of Genes and Genomes (KEGG ) ortholog (KO) matches, the most abundant metabolic categories are carbohydrate, lipid, aminoacid, and glycan biosynthesis. When it comes to the processing of genetic information, the most abundant genes have functions assigned to protein folding, sorting, and degradation. Signal transduction pathways are the most abundant category of all with 1,717 transcripts. Transport and catabolism, cell growth and death, and cellular community are the most abundant categories within cellular processes. Finally, the most important organismal systems in the slug are the endocrine, immune, nervous, and digestive system, followed by development and regeneration. Within this last group, *D. laeve* has 42 KO related to axon regeneration, raising the possibility that the slug can regenerate its nervous system. Also within the development and regeneration category, the *D laeve* genome contains 33 KO related to osteoclast differentiation. Key proteins such as colony stimulating factors, interleukins, and RANKL are expectedly absent in the slug. However, some interesting KOs present are calcitonin receptor, microphthalmia-associated transcription factor, sequestosome, and cathepsin K, as well as other proteins that relate osteoclast differentiation to the immune system (Takayanagi 2007; Lorenzo et al. 2008; Tsukasaki and Takayanagi 2019).

**Fig. 5.**
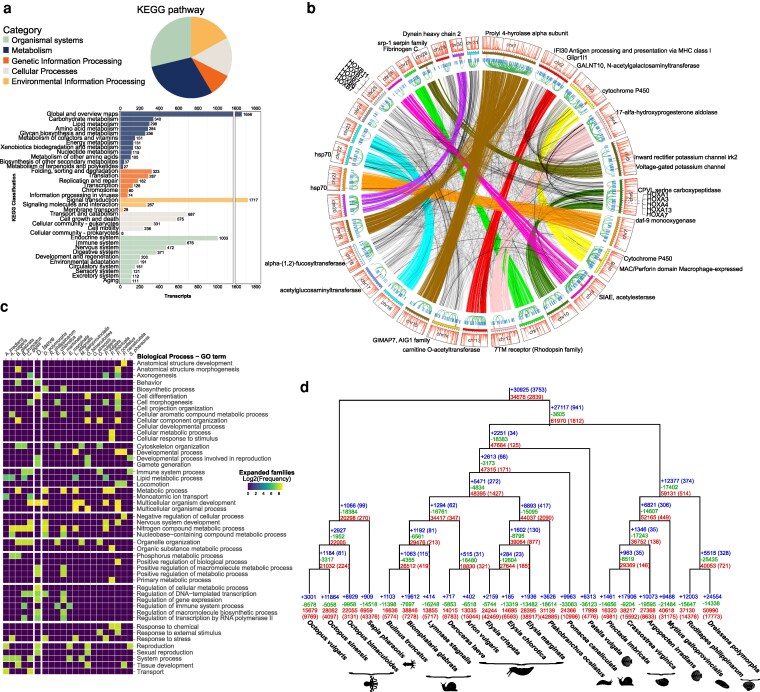
Functional overview of the *D. laeve* proteome and evolution within mollusks via gene duplication and gene loss. a) Proportions and number of transcripts assigned to 5 categories according to their KEGG annotation: Organismal Systems, Metabolism, Genetic Information Processing, Cellular Processes, and Environmental Information Processing. b) Central circle area: Syntenic relationships between chromosomes revealed by close paralogy connections of genes annotated by eggNOG Mapper. Stronger interchromosomal connections highlight notable correspondences. Rings from inside-out: Intrachromosomal arches connect paralogous genes that are more than 5 Mb apart. Smaller arches connect paralogous genes located less than 5 Mb apart (most are so close to each other that only a tick can be observed). Solid notches in the following outer ring mark clusters of 5 or more related genes within segments of 5 Mb. Solid bars along the circumference represent chromosomes and tickmarks indicate intervals of 5 Mb. The outer-most ring indicates the abundance of telomeric motifs as in Fig. 3 and the chromosome number. The eggNOG functional descriptions of some genes are provided in the outer region along with the location of the Hox gene clusters in chromosomes 6 and 25. c) Frequency of the top GO terms associated with the expanded gene families found in a CAFE analysis of mollusk proteomes. d) Reconciliation tree depicting inferred protein gains and losses in extant mollusk species and their ancestors. Numbers in parentheses at speciation events indicate *de novo* generated proteins while those at the leaf level represent proteins with no identified orthologs.

The 2 KO groups with the highest number of members are the C1q-related factor and the acetylcholine nicotinic receptor (Fig. 5a). The latter have been proposed to serve special functions in the nervous system of *mollusks* (van Nierop et al. 2005; Albertin et al. 2015).

The distribution of protein-coding orthologs in chromosomes confirms the interchromosomal similarity pattern shown in Fig. 1c, consistent with the above mentioned WGD in *Stylommatophora* (Chen et al. 2022). Thus, most chromosomes have at least one clear cognate, that is, a homologous chromosome in the same species resulting from WGD (Supplementary Table S7, Fig. S3). For example, chromosome 4 is cognate to chromosome 12, chromosome 7 to 21, and chromosome 20 to 30. However, note that chromosome 1 is cognate to both chromosomes 15 and 19, but the latter are not cognate to each other. This suggests that the fusion of the cognates of 15 and 19 formed 1 or the fission of 1 yielded 15 and 19 (Fig. 5b). Hence, the genomic distribution of putative ohnologs seems to fit the WGD model followed by chromosome fusions and fissions. The distribution of tandem repeats containing the consensus telomeric motif TTAGGG in interstitial telomeric sequences also reflects this history.

Intrachromosomal homology relationships can also reveal segmental duplications, when the 2 presumptive paralogs are found in close proximity. Analyzing the distribution of the paralogs, we found 720 possible segment duplications containing protein-coding genes (Fig. 5b). Extending the analysis, we identified clusters of homologous genes with at least 5 closely related genes located within a 5 Mb range. These clusters are present on almost all chromosomes, and some of them could predate genome duplication such as those of cytochrome P450 present on chromosomes 10 and 4. Our study also identified putative gene clusters, many of which are involved in defense against harmful external stimuli and could be the target of further research.

We also found clusters of genes related to AIG1 and hsp70 (Fig. 5b), which have been implicated in adaptation to thermal stress in mollusks and nematodes (Guerin et al. 2019). Other genes are involved in defense against pathogens (Fibrinogen, Serpin, MPEG1), matrix remodeling, or sterol metabolism. A cluster on chromosome 18 contains genes annotated as fucosyltransferase. Interestingly, this protein is one of the main protein components of slug mucus (Yuan et al. 2025).

The largest group by number of genes is located on chromosome 5. It contains voltage-gated potassium channel subunits, involved in the control of cell proliferation and apoptosis (Bachmann et al. 2020). Furthermore, a cluster of Glipr1l1 proteins, also present in humans and mice and involved in sperm-oocyte interaction (Ren et al. 2006; Gibbs et al. 2010) was found. Intriguingly, the most common functions and GO terms found in expanded gene families of *D. laeve* include sexual reproduction, gamete formation, the endocrine system and the metabolism of steroid hormones (Supplementary Table S8).

We ran REvolutionH-tl to find ortholog groups among the set of proteins of 21 species of *mollusks* from the classes *Cephalopoda, Gastropoda, and Bivalvia* (Table 1). We then used CAFE to model the process of gene birth and death with phylogenetic information. We found 27 fast-evolving families in the *D. laeve* lineage (Supplementary Table S9). Contracted families are associated with GO terms for acetyltransferase activity, G-protein coupled receptor, collagen and actin binding, and hydrolase activity. Expanded families are associated with the regulation of behavior, response to stress in the endoplasmic reticulum and mitochondrion, and RNA biology.

We also investigated expanded gene families in general. From this analysis, the most common GO terms in them are related to development, biosynthetic and metabolic processes, gene regulation, and, intriguingly, sexual reproduction and gamete development (Fig. 5c). Phylogenetic modeling of the pattern of ortholog group changes shows a pattern of gene expansions in the cephalopod lineages. Meanwhile, most ortholog groups in gastropods show a reduction in their members both in extant groups and in ancestors showing a dynamic landscape of gene evolution Fig. 5d). This reflects the highly diverse morphologies and habitats in mollusks, such as kleptoplasty of *plakobranchidae*. In the case of *Stylommatophora*, rapid gene loss could have followed the whole genome duplication event that made most of the genome redundant.

### Small RNA from RNA silencing pathways

**Fig. 6.**
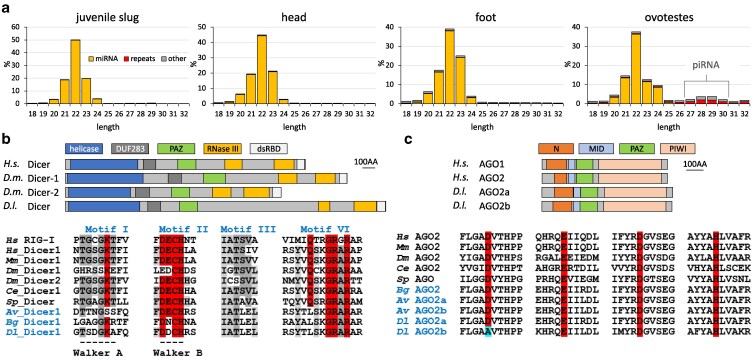
Analysis of RNA-silencing pathways in *Deroceras laeve*. a) Small RNAs from different organs. Plots show frequencies of 18–32 nucleotide RNAs sequenced from whole juvenile slugs, as well as head, foot, and ovotestes from adult animals. Each plot represents an average of 3 biological replicates. b) Dicer-dependent small RNA biogenesis relies on a single Dicer homolog with canonical domain organization. Below the domain organization, the motifs important for ATP-dependent helicase activity implicated in processive long dsRNA cleavage are highlighted in red (Kidwell et al. 2014). Species name initials: Av, *Arion vulgaris*; Bg, *Biomphalaria glabrata*; Ce, *Caenorhabditis elegans*; Dl, *D. laeve*; Dm, *Drosophila melanogaster*; Hs, *Homo sapiens*; Mm, *Mus musculus*; Sp, *Schizosaccharomyces pombe*. c) Analysis of AGO-clade Argonaute proteins. Two AGO protein-coding genes were found in the *D. laeve* genome assembly. Both proteins have the typical AGO protein architecture and their closest homolog in other species is AGO2. Hence, they were denoted AGO2a and AGO2b. Below the domain composition, the conservation of the 4 amino acid residues in the PIWI domain that form the “catalytic tetrad” in the functional nuclease is highlighted by shaded boxes (Nakanishi et al. 2012).

Small RNAs in RNA silencing pathways were identified from juvenile slugs and adult foot, head, and ovotestes (Fig. 6a). This analysis revealed that the most abundant small RNA type in all samples was microRNA (miRNA), while in ovotestes we also observed PIWI-associated RNA (piRNA). The levels of putative small interfering RNAs (siRNAs) from the endogenous RNA interference pathway (RNAi) were negligible and the 21–23 nt RNA population was strongly dominated by miRNAs (Fig. 6a).

This result was consistent with the analysis of Dicer, an RNase III producing small RNAs from the RNAi and miRNA pathways. Dicer of *D. laeve* has the organization of the canonical domain (Fig. 6b). Its coding sequence is longer than that of human or *D. melanogaster*. However, this difference appears to involve only unstructured parts of the enzyme. The N-terminal helicase domain of Dicer is important for substrate recognition and its ATPase activity is important for processive cleavage of long double-strand RNA into siRNAs (Kidwell et al. 2014). Consistent with the dominance of miRNAs and the scarcity of siRNAs in the sequenced samples, mutations were found in conserved segments of the Dicer helicase domain (Fig. 6b) known to be relevant for processive cleavage (Kidwell et al. 2014).

Two genes were identified in the assembled genome encoding Argonaute protein homologs (AGO), which bind to the small RNAs produced by Dicer (Fig. 6c). They have the canonical organization of the AGO domain and their closest homologs annotated in other genomes were AGO2 proteins. *D. laeve* AGO2 proteins differed primarily in their 60–70 amino acid long N-terminal proline rich region. Furthermore, the amino acid residues of the catalytic tetrad required for endonucleolytic activity (Nakanishi et al. 2012) are conserved in AGO2a, thus predicted to be “slicer” (Fig. 6c). AGO2b carries a point mutation in one of the tetrad amino acid residues and is likely not to cleave its targets (Fig. 6c, Supplementary File S6). These results suggest an organization of the small RNA pathways of *D. laeve* similar to that of mammals, in which a single Dicer protein mainly generates miRNAs and the piRNA pathway is present primarily in the germline.

### miRNA annotation

Micro RNAs were predicted using the MIREAP miRNA predictor and the loci that give rise to miRNAs were individually inspected and curated to produce a reliable list of miRNA genes (Supplementary Table S1). We identified 177 miRNA-encoding loci with high and moderate confidence. They were compared with miRNAs of the *bivalve Pacific oyster* (*Magallana gigas*) and the *gastropods owl limpet* and *red abalone* (*Haliotis rufescens*) identifying 5 miRNA families absent in the *D. laeve* annotation (Supplementary Table S2). Of these, only miR-34 could be identified and annotated by additional small RNA data analysis and was added to the list of annotated miRNAs (Supplementary Table S1). Thus, altogether, we annotated 178 pre-miRNA loci. Of them, 55 encoded a mature miRNA that perfectly matches an already annotated miRNA in miRGeneDB 3.0. In addition, 72 precursor loci are predicted to produce mature miRNAs matching nucleotides 2 to 10 of annotated miRNAs, and an additional 6 to produce miRNAs matching seed nucleotides 2 to 8 of known miRNAs. In general, these 133 miRNAs match 50 families found from *Eumetazoa* to *Gastropods* (Supplementary Table S2). Interestingly, for many miRNAs, *D. laeve* has twice as many copies relative to *Haliotis rufescens* and *Lottia gigantea*, which is also consistent with stylomatophoran whole genome duplication (Chen et al. 2022). Of the remaining 45 novel precursor loci, 25 and 20 were considered of high and moderate confidence, respectively, while 14 were paralogs of 6 different miRNAs (Supplementary Table S1).

Small RNA analysis also allowed the identification in *D. laeve* of miRNAs that make up 95% of the 21–23 nt RNAs (Fig. 6a) and revealed more miRNA loci than in other gastropods present in miRGeneDB. However, this increase appears to be primarily caused by chromosomal level duplication events as the number of miRNA families remains constant. Hence, this does not represent a major expansion in the miRNA repertoire as observed in cephalopods (Zolotarov et al. 2022). The adaptation of Dicer for miRNA biogenesis is consistent with the profile of small RNA and the sequence changes in the helicase domain (Fig. 6a, b). In fact, ancestral protein reconstruction suggests that the helicase domain in the lineage leading to mollusks shows signs of deterioration of its role in RNAi (Aderounmu et al. 2023).

Although negligible levels of endo-siRNAs and the Dicer helicase domain sequence imply inefficient or absent RNAi, long dsRNA delivery has been reported to induce RNAi in several gastropods, including *Limnaea stagnalis* (Fei et al. 2007; Guo et al. 2010) and *Biomphalaria glabrata* (Jiang et al. 2006; Knight et al. 2011). Since *Biomphalaria* The Dicer helicase exhibits amino acid changes similar to those of *D. laeve* (Fig. 6b), the ability or inability of long dsRNA to induce RNAi in *D. laeve* needs to be tested experimentally.

### piRNA pathway and piRNA clusters

**Fig. 7.**
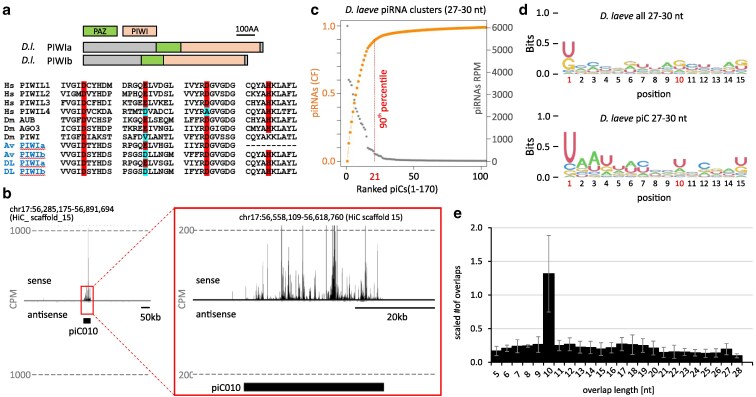
Analysis of the piRNA pathway in *Deroceras laeve*. a) Two PIWI protein-coding genes were found in the *D. laeve* genome assembly. Both proteins have the typical PIWI protein architecture and are considerably divergent (BLAST alignment shows 41% identity and 61% similarity). Below the domain composition, conservation of the 4 amino acid residues in the PIWI domain that form the “catalytic tetrad” is highlighted in shaded boxes. PIWIa has all residues conserved, PIWIb carries one E>D aminoacid substitution. b) A representative genome track depicting a typical unidirectional piRNA cluster (piC10) with 27–30 nucleotide small RNAs mapped to it. The 27–30 nucleotide reads mapping to the + (sense) and − (antisense) strands are shown separately. CPM, counts per million. c) Ranked annotated piRNA clusters displaying the cumulative fraction (CF) of piRNAs. The top 21 piRNA clusters ranked by reads per million (RPM) represent 90% of piRNAs mapping to all clusters. d) A sequence logo of all 27–30 nucleotide reads mapped to the genome (top) and reads mapping to annotated piCs (bottom). A typical 1U pattern is observed for the entire 27–30 nt RNA population and the signal is increased when only considering reads mapping to piCs. The 10A signature typical of the ping-pong mechanism is not apparent. e) Analysis of 27–30 nucleotide RNA overlaps. The most common overlap is for 10 nucleotides suggesting that a fraction of piRNAs originates from piRNA-mediated cleavage, although this fraction is not large enough to manifest as a 10A signature in the sequence logo.

On the contrary, the piRNA pathway in *D.laeve* is relatively simple. Two genes encoding piRNA-binding PIWI protein homologs with the usual domain organization were identified in the assembled genome (Fig. 7a, Supplementary File S6). All critical amino acid residues required for endonucleolytic activity are conserved in one of them, the PIWIa protein (Fig. 7a). The second predicted protein, PIWIb, carries an E>D aminoacid substitution in a critical catalytic residue, but its effect on nuclease activity remains to be determined (Fig. 7a). Therefore, PIWIa is predicted to be a “slicer”, while the nuclease activity of PIWIb is uncertain. From the genome sequence, it could not be inferred whether any of the PIWI proteins are involved in transcriptional silencing.

The small RNA size distribution suggests that piRNAs are 27 to 30 nucleotides long with the main peak at 29, while more than half of these small RNAs are repeat derived (Fig. 6a). In total, we annotated 170 primary clusters producing piRNAs in ovotestes (Supplementary Table S3, Fig. 3b). When ranked by number of piRNAs produced, the first 21 unidirectional clusters contribute 90%, thus representing the main primary source (Fig. 7b, c). This is similar to *Arion vulgaris*, in which the top 19 clusters contribute 90% of piRNAs (Konstantinidou et al. 2024).

Sequence analysis of the 27 to 30 nucleotide population yielded the typical 1U signal, which became stronger when only reads mapping to piC were considered (Fig. 7b, c). At the same time, the 10A signature typical of the ping-pong piRNA amplification mechanism (Aravin et al. 2007; Brennecke et al. 2007; Gunawardane et al. 2007) was not apparent in the sequence logo, suggesting a low or no contribution of target cleavage by 1U primary piRNAs to the biogenesis of additional piRNAs. However, a detailed analysis of the overlap length distribution between piRNAs showed that a 10-nucleotide overlap is more frequent than others (Fig. 7e), suggesting that there might be a small population of secondary piRNAs, which also resembles *A. vulgaris* (Konstantinidou et al. 2024). In line with the somatic piRNAs in other mollusks (Jehn et al. 2018), the piRNA pathway in *D. laeve* appears to be active in somatic organs, as indicated by the expression of both PIWI genes and the low but detectable presence of 27 to 30 nt piRNAs in head and foot.

### Hox clusters

**Fig. 8.**
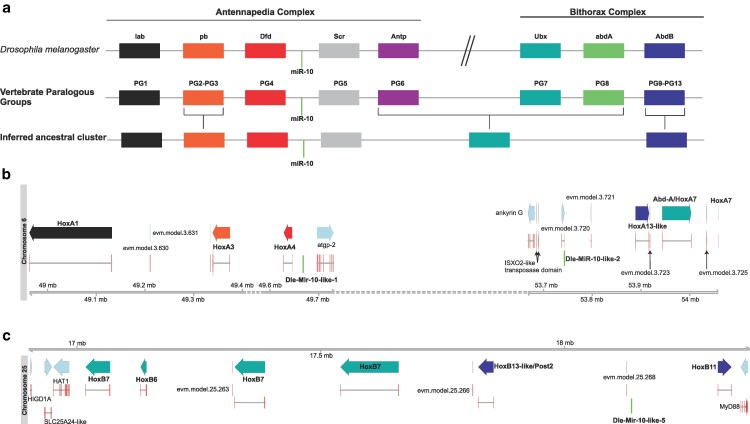
Hox clusters of *D. laeve*. a) Structure of Hox clusters in *Drosophila* and Vertebrates. *Drosophila* has an Antenappedia and a Bithorax complex. Vertebrates possess in general 4 clusters with Paralogous Groups (PG) 1 to 13, equivalent to the *Drosophila* genes. PG2 and PG3 are orthologous to *pb* and PG9-PG13 are orthologous to AbdB. The inferred ancestral Hox cluster had 6 genes. Both *Drosophila* and vertebrate clusters possess a conserved miR-10. Adapted from (Mallo and Alonso 2013). b) *D. laeve* HoxA cluster in chromosome 6 has putative orthologs of the first 3 ancestral PGs and a miR-10-like locus in the expected place in comparison to *Drosophila* and vertebrates. The second part of this cluster, which is located after approximately 3 Mb on the same chromosome, contains possible derivatives of PG7-8 and another copy of miR-10 upstream of these genes. c) *D. laeve* HoxB cluster in chromosome 25 seems to contain only bithorax “posterior” genes from PG6-13. Another copy of miR-10-like miRNA is present within this cluster.

We found 2 Hox clusters, each with 6 genes present on cognate chromosomes 6 and 25 that we named HoxA and HoxB, respectively (Fig. 8a). Cluster A has orthologs of the first 3 paralog groups of the ancestral Hox cluster separated by 3 Mb from the other 3 Hox genes transcribed in the opposite orientation, which correspond to the Bithorax complex (PG7-13) (Fig. 8b). A miR-10-like miRNA is located after the third gene of the cluster, similarly to both *Drosophila* and vertebrate Hox clusters and another copy preceding the last 3 HoxA genes (Fig. 8b). Cluster B appears to contain 6 Bithorax or “posterior” genes from PG6 to 13, descendants of the last 2 ancestral Hox genes. The last gene of this cluster (HoxB11) is inverted relative to the rest of the HoxB genes and a copy of miR-10-like precedes it. Hence, with a conserved cluster organization, these Hox genes in *D. laeve* have undergone unique rearrangements.

### Conclusions

The genomic resources provided here, along with the increasing availability of high-quality genomes of related species, provide unique opportunities to study whole genome duplication and retrotransposon expansion in the context of evolution, sexual dynamics, population isolation, and speciation (Otto 2007). The ubiquitous garden slug *Deroceras laeve* has the potential to become a model organism in the tradition of biology workhorses such as *Drosophila*, *mouse*, and *chicken*. In support of this, we have recently published a histological atlas that describes the anatomy of this species, its ability for degrowth and regrowth, and morphological aspects of the regeneration of its tail (Lozano-Flores et al. 2024). This work now makes its full genome and extensive annotation fully accessible, which constitutes another step in facilitating its use as a model to study regeneration, RNA biology, and genome evolution.

## Data Availability

**Accession codes:** Raw DNA-seq and RNA-seq data were deposited in the SRA-NCBI database under BioProjects PRJNA1035784, PRJNA1213857, and PRJNA1237345. The final genome assembly has been deposited at DDBJ/ENA/GenBank under the accession JBNOKR000000000. The version described in this paper is version JBNOKR010000000. Supplementary Data and genome assembly is available at FigShare (https://doi.org/10.25387/g3.29429681).
